# Shifts in subsets of CD8+ T-cells as evidence of immunosenescence in patients with cancers affecting the lungs: an observational case-control study

**DOI:** 10.1186/s12885-015-2013-3

**Published:** 2015-12-28

**Authors:** Oscar Okwudiri Onyema, Lore Decoster, Rose Njemini, Louis Nuvagah Forti, Ivan Bautmans, Marc De Waele, Tony Mets

**Affiliations:** 1 Gerontology Department and Frailty in Aging Research (FRIA) Group, Faculty of Medicine and Pharmacy, Vrije Universiteit Brussel, Laarbeeklaan 103, B-1090 Brussel, Belgium; 2 Department of Medical Oncology, Oncologisch Centrum, Universitair Ziekenhuis Brussel & Vrije Universiteit Brussel, Laarbeeklaan 101, B-1090 Brussel, Belgium; 3 Department of Geriatrics, Universitair Ziekenhuis Brussel, Laarbeeklaan 101, B-1090 Brussel, Belgium; 4 Laboratory of Hematology, Universitair Ziekenhuis Brussel, Laarbeeklaan 101, B-1090 Brussel, Belgium

**Keywords:** Cellular senescence, Immunosenescence, Lung cancer, Chemotherapy, Immune risk profile

## Abstract

**Background:**

Shifts in CD8+ T-cell subsets that are hallmarks of immunosenescence are observed in ageing and in conditions of chronic immune stimulation. Presently, there is limited documentation of such changes in lung cancer and other malignancies affecting the lungs.

**Methods:**

Changes in CD8+ T-cell subsets, based on the expression of CD28 and CD57, were analysed in patients with various forms of cancer affecting the lungs, undergoing chemotherapy and in a control group over six months, using multi-colour flow cytometry.

**Results:**

The differences between patients and controls, and the changes in the frequency of CD8+ T-cell subpopulations among lung cancer patients corresponded to those seen in immunosenescence: lower CD8-/CD8+ ratio, lower proportions of CD28+CD57- cells consisting of naïve and central memory cells, and higher proportions of senescent-enriched CD28-CD57+ cells among the lung cancer patients, with the stage IV lung cancer patients showing the most pronounced changes. Also observed was a tendency of chemotherapy to induce the formation of CD28+CD57+ cells, which, in line with the capacity of chemotherapy to induce the formation of senescent cells, might provide more evidence supporting CD28+CD57+ cells as senescent cells.

**Conclusion:**

Immunosenescence was present before the start of the treatment; it appeared to be pronounced in patients with advanced cases of malignancies affecting the lungs, and might not be averted by chemotherapy.

## Background

Unfavourable shifts in subpopulations of T-cells, resulting in a decreased CD4+/CD8+ ratio and in the accumulation of senescent and terminally differentiated T-cells [1–4], as part of immunosenescence are widely observed in human aging [5, 6]. Premature or more pronounced signs of immunosenescence, known as an immune risk profile (IRP), have been documented in chronic disorders like rheumatoid arthritis [7, 8] and chronic heart failure [9], as well as in persistent viral infections with cytomegalovirus (CMV) [10, 11] and human immunodeficiency virus (HIV) [12, 13]. In all the above situations, immunosenescence was associated with negative outcomes such as the degeneration of biological structures, enhanced disposition to new infections and appearance of new pathological conditions, treatment failure, and increased mortality [6, 14–17]. In consideration of the long carcinogenesis period needed for cancer development and progression, and the prolonged immune stimulation that is associated with cancer progression, a potential role for immunosenescence in cancer has been suggested; however, strong evidence in support of this hypothesis is still lacking [18, 19]. At the moment, some indications linking immunosenescence parameters to cancer have emerged [20–23]. Nevertheless, the senescent T-cells that are known to accumulate during immunosenescence have not been well explored in cancer. Also, little information is available to relate cancer disease stages to changes in the level of senescent T cells and other shifts in subpopulations of T-cells that characterize immunosenescence.

In vitro studies have shown that the occurrence of cellular senescence is enhanced by DNA damaging chemotherapy [24, 25]. This stress induced premature senescence (SIPS) [26, 27] has not been well documented in vivo, where it was mainly explored in cancer cells and in the tumour microenvironment [28]. DNA damaging chemotherapy, when administered in vivo, will however, also affect other cells in the body, including T-lymphocytes [29, 30]. Senescent T-cells have been phenotypically described by their loss of CD28 expression [31], and/or the expression of CD57 [1, 3]. Others and our group have shown that the expression of CD57 (found on both CD28-CD57+ and CD28+CD57+ cells) was associated with pronounced characteristics of senescent cells such as loss of proliferation capacity in vitro, telomere attrition, increased expression of cyclin dependent kinase (CDK) inhibitors – p16 and p21, and the higher presence of these cells in elderly than in young humans [1–3, 32]. The cells also showed a cytokine secretion profile analogous to the senescence associated secretary phenotypes [1, 33, 34]. CD28+CD57+ and CD28-CD57+ cells were found to have different homing and differentiation characteristics, which might point to a different origin for both senescent phenotypes [32]. While the CD28-CD57+ cells, also considered as terminally differentiated effector memory cells, and the CD28-CD57- cells, considered as effector memory cells, might not provide good anti-tumour immunity but more adverse effects, the CD28+CD57- cells, because of their enrichment with naïve and central memory cells, and their characteristic homing to secondary lymphoid organs, would provide better immunity against cancer [1, 32, 35]. Other attributes of the four subpopulations, including their cytokine secretion profile, proliferation capacity, differentiation characteristics, expression of exhaustion markers, expression of survival markers, expression of senescence markers, and apoptotic tendency have been previously determined and were used in the classification of the four subpopulations [1, 3, 32, 36].

Lung cancer is one of the most devastating cancers and the leading cause of cancer deaths worldwide [37, 38]. More than 65 % of people diagnosed with lung cancer are at least 65 years old [37–39], making it a disease that is predominant in older people. Emerging evidence indicates that immune markers might allow stratification of lung cancer prognosis [40]. Recently, post chemotherapy T-cell recovery, linked with enhanced CD8+ T-cell proliferation, was described as a good prognostic factor for patients with various forms of lung cancer [41]. A related report showed an increase in the in vitro proliferation of CD8+ T cells from malignant mesothelioma (MM) and non-small cell lung cancer (NSCLC) patients compared with healthy controls [42]. This study, however, did not consider the impact of different subpopulations of CD8+ T-cells, which are known to have different proliferation capacities [1, 34].

In the present study, we hypothesized that malignancies of the lung would be associated with shifts in CD8+ T-cells related to immunosenescence, including an increased frequency of senescent subpopulations of CD8+ T cells that would be at least similar to the elderly values, and which might be enhanced with disease advancement. We also hypothesized that chemotherapy would modulate the formation of senescent cells. These hypotheses were tested through a longitudinal observation of lung cancer patients undergoing chemotherapy and a control group comprising older normal persons.

## Methods

### Participants

A cohort of patients with various malignancies affecting the lungs, mostly lung cancer patients scheduled to undergo chemotherapy and a cohort of community dwelling, normal older persons as controls were prospectively recruited from the Belgian Caucasian population into the study at the Universitair Ziekenhuis Brussel, and each participant was followed up for six months between November 2011 and July 2013. The exclusion criteria for all participants included the presence of haematological disorders and/or prior immunodeficiency, and involvement in strenuous exercise within 24 h to the sampling [43, 44]. The control group passed a comprehensive medical assessment before they were included in the study. The participants were sampled at baseline (T0), after which the patients started receiving chemotherapy, at one month (T1), three months (T3), and at six months (T6). The study was approved by the Institutional Review Board of the Universitair Ziekenhuis Brussel (OG016) and all participants provided written informed consent.

### Blood sample collection, enumeration and preparation

Peripheral venous blood samples from the participants were collected in EDTA tubes, and processed immediately. The enumeration of blood cells was done in a Cell Dyn Sapphire^®^ Analyzer (Abbot Diagnostics, Wavre, Belgium). Peripheral blood leukocytes (PBL) were obtained by incubating portions of the blood samples in an ammonium chloride-based lysis buffer for 10 min to lyse the red blood cells. The resulting mixture was centrifuged at 2800 rpm for 4 min to obtain the PBL, which were washed in 1 % BSA-PBS and used for analysis of the cell surface markers and delineation of the different subpopulations.

### Flow cytometry analysis

PBL from the subjects were surface-stained with a panel of antibodies. Briefly, about 5 × 10^5^ lymphocytes in 50 μl of 1 % PBS-BSA were incubated with 20 μl of appropriate combination of antibodies for 20 min at room temperature in the dark. Then, the cells were washed with PBS and were resuspended in 500 μl of PBS for flow cytometric analysis. For all samples, 100,000 PBL events were acquired for analysis in a five-colour flow cytometer (Cytomics FC 500) (Beckman Coulter, Analis, Belgium). The following antibodies were used in appropriate combinations and concentrations: PE-cy5-anti-CD8 (BD Biosciences, Erembodegem, Belgium), FITC-anti-CD28, PE-anti-CD57 and PE-cy7-anti-CD3 (Biolegend, Imtec, Belgium). All antibodies were matched with isotype controls (Santa Cruz Biotech, Heidelberg, Germany). Quality control panels were used in order to exclude autofluorescence, fluorochrome interferences and dead cells; including compensation controls based on data collected from single fluorochrome staining, fluorescence–minus-one controls that includes other stains and exclude the stain in a particular channel to define the boundary between positive and negative cells in a given channel, and dead cell exclusion control using 7-amino actinomycin-D (7-AAD) staining. Also, quality controls for the machine were performed daily by checking the detector voltage values for conformity with initial protocol and running daily verification of the dynamic range of the detectors using standardized quality control compensation beads.

**Fig. 1:**
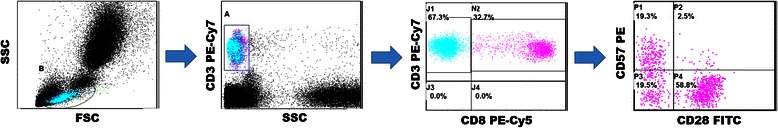
Representative dot plots for the delineation of the different subpopulations by flow cytometry, By combining side (SSC) versus forward scatter (FSC), and CD3 fluorescence versus SSC plots, CD3+ cells were identified. CD8+ cells were obtained from the pure CD3+ population, and were further subdivided based on the expression of CD28 and CD57

The different subpopulations of CD8+ T-cells were delineated as we previously described [3, 32] and shown in Fig. 1. The T-lymphocytes were identified and gated using a combination of light scatter parameters (forward scatter and side scatter) and fluorochrome conjugated anti-CD3 antibody fluorescence, following fluorescent antibody labelling of PBL. Next, the CD8+ T-lymphocytes were gated within the T-cells (CD3+ lymphocytes). Flow cytometry dot plots were used to separate and identify different subpopulations of CD8+ T-lymphocytes based on their expression of CD28 and CD57.

### Statistical analysis

Statistical analysis was performed using SPSS (version 22). The primary outcome measures, which are data on immunological parameters are presented in dot plots, with the bottom and top of the boxes representing the lower (Q1) and upper (Q3) quartiles respectively, the dark band inside the box representing the median, and the whiskers representing the highest and lowest observed values that were not outliers. The baseline ages are reported as median with Q1-Q3 in brackets. Differences in evolution of the outcome variables among independent groups were analysed with the Kruskal-Wallis test. Between two groups analysis was performed using the Mann-Whitney U test. Evolution of outcome measures over time was analysed using the Friedman’s test. When the evolution over 6 months was significant, differences between the baseline and subsequent time-points were analysed with the Wilcoxon Rank test. Changes between two points in different groups were compared using the Mann-Whitney U test. The above statistical descriptions also applied to the following: (i) the analysis of the data sets with or without participants that withdrew at some point during sampling in order to exclude the impact of participant withdrawal; (ii) the exclusion of possible effects of radiotherapy on the treatment outcome by analysing all patients together, and then excluding those treated with a combination of chemotherapy and radiotherapy; (iii) sex bias exclusion among the cancer patients and controls by analysing the data according to sex before pulling the data together. Exact statistical testing was used in the estimation of significant differences. Differences were considered to be significant at two-sided *p* < 0.05.

## Results

Twenty four patients with various malignancies affecting the lungs (60 y (56–66); 20 males, 4 females) receiving platin-based chemotherapy were included in the study before the start of their treatment, as well as 28 community-dwelling healthy older persons (72 y (68–74); 11 males, 17 females). The sample sizes we used have been proved sufficient in other related studies [3, 45–47]. The stage IV patients (58 y (55-63)), but not the stage III patients 66 y (56–76) had a significantly lower age than the controls (*p* < 0.001). Diagnosis details and treatment schedules are listed in Table 1. To exclude sex bias, observations among the cancer patients and controls were also analysed according to sex. As these comparisons did not significantly differ from those of all cancer patients with the whole control group, we do not report sex comparisons. As some patients received concomitant radiotherapy (stage III SCLC patients and all patients that received cisplatin-docetaxel chemotherapy), we also analysed our data for any possible influence of radiotherapy but could not find any significant effect attributable to radiotherapy. This permitted us to merge all patients under chemotherapy (with or without radiotherapy) together in this report. Since a few patients died during the study, we also analysed the results by excluding patients that did not complete all four samplings. The number of patient withdrawals was small and did not influence the outcome.

**Table 1:** The distribution of various cancers of the lung among the patients and the treatment regimens they received

Sex	Subtype 1	Subtype 2	Stage	Treatment (N)	N	N	N	N
					T0	T1	T3	T6
Male	SCLC		IIIA	CE + R (1)	1	1	1	0
	SCLC		IIIB	CE + R (1)	1	1	1	1
	SCLC		IV	CE (1)	1	1	1	1
	MM		IV	CP (2)	2	2	2	1
	NSCLC	NSCC	IIIA	CD + R (1)	1	1	1	1
		NSCC	IV	CD + R (1), CP (11)^a^	12	12	10	8
		SCC	IIIB	CD + R (1), CG (1)	2	2	2	2
Female	NSCLC	NSCC	IIIA	CV (1)	1	1	1	1
		NSCC	IIIB	CP (1)	1	1	1	0
		NSCC	IV	CP (2)	2	2	2	2

Lung cancer: *SCLC* small cell lung cancer, *MM* mesothelioma of the lung, *NSCLC* non-small cell lung cancer, *SCC* squamous cell carcinoma of the lung, and *NSCC* Non squamous cell carcinoma

Treatment: *CD* cisplatin & docetaxel, *CE* cisplatin & etoposide, *CG* cisplatin & gemcitabine, *CP* cisplatin & pemetrexed, *CV* cisplatin & vinorelbine, *R* radiotherapy

*N* Number of recipients for a particular chemotherapy regimen

^a^The number of participants reduced to 10 and 8 at the 3^rd^ and 6^th^ months respectively

**Fig. 2:**
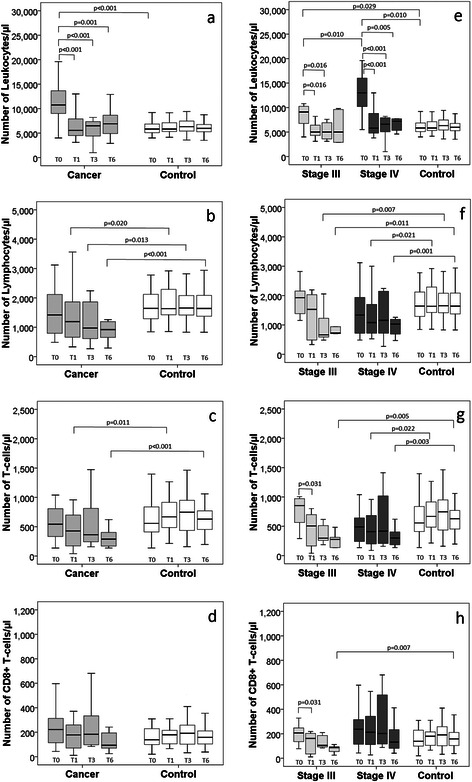
The absolute numbers of leukocytes, lymphocytes, T-lymphocytes and CD8+ T-lymphocytes among lung cancer patients and controls (**a**–**d**) and according to cancer disease stages (**e**–**h**), at baseline (T0), 1 month (T1), 3 months (T3), and 6 months (T6)

Figure 2 shows the absolute numbers of leukocytes, lymphocytes, T-lymphocytes and CD8+ T-lymphocytes at the various sampling points in all participants, and after stratifying the cancer patients according to disease stages. At baseline, the leukocyte numbers were significantly higher in the cancer patients than in the controls, also after separating the cancer patients according to the disease stages (Fig. 2a & e); however, the stage IV cancer patients had significantly higher leukocyte numbers than their stage III counterparts (Fig. 2e). At baseline, lymphocyte (Fig. 2b & f), T-lymphocyte (Fig. 2c & g), and CD8+ lymphocyte (Fig. 2d & h) concentrations were not different between the patients and controls. During follow-up, there was a decline in the cell counts among the cancer patients, which returned to levels similar to the control group in leukocytes, and levels lower than the controls among lymphocytes and T-lymphocytes. The CD8+ T-cells remained similar in the lung cancer group and controls.

**Fig. 3:**
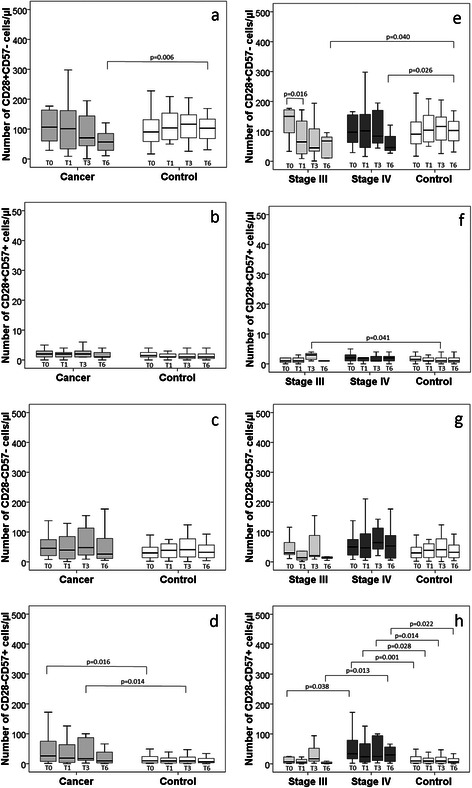
The absolute numbers of CD28+CD57-, CD28+CD57+, CD28-CD57-, and CD28-CD57+ cells, among lung cancer patients and controls (**a**–**d**) and according to cancer disease stages (**e**–**h**), at baseline (T0), 1 month (T1), 3 months (T3), and 6 months (T6)

Figure 3 shows the absolute numbers of the CD8+ subpopulations CD28+CD57-, CD28+CD57+, CD28-CD57- and CD28-CD57+ among the participants, including the cancer stages, over the six months period. The absolute numbers of the CD28+CD57- (Fig. 3a & e), CD28+CD57+ (Fig. 3b & f), and CD28-CD57- cells (Fig. 3c & g) were similar in the cancer patients and controls, even after stratifying the cancer patients based on disease stages, except at the 6^th^ month, where the CD28+CD57- cell counts were lower among the lung cancer patients than the controls, and the 3^rd^ month, in which the absolute number of CD28+CD57+ cells was higher among stage III cancer patients than the controls. A different scenario was observed among CD28-CD57+ cells (Fig. 3d & h); the absolute cell count remained higher among the cancer patients, mainly among the stage IV patients, compared with the controls. Also, the CD28-CD57+ cell count among the stage IV cancer patients at baseline was significantly higher than among the stage III patients. The frequency of CD28-CD57+ cells among the stage III patients remained similar to the controls at all time-points.

The evolution of cells, over 6 months did not differ among the subpopulations apart from the stage III lung cancer patients, for whom the frequency of CD28+CD57- cells at baseline was significantly higher than the frequency after one month. In addition, the change in number of CD28+CD57- and CD28-CD57- cells between the baseline and one month reflected a significant decrease among stage III patients compared with the controls (all *p* < 0.005).

**Fig. 4:**
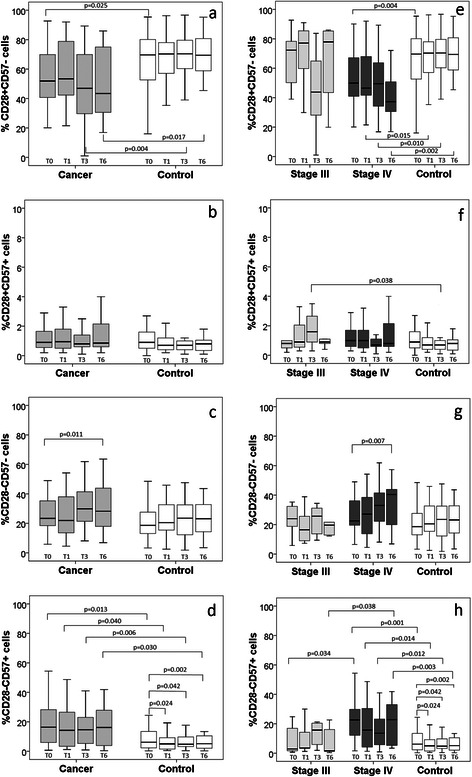
The proportions of CD28+CD57-, CD28+CD57+, CD28-CD57- and CD28-CD57+ cells from CD8+ T-cells, among lung cancer patients and controls (**a**–**d**) and according to cancer disease stages (**e**–**h**), at baseline (T0), 1 month (T1), 3 months (T3), and 6 months (T6)

The differences in the evolution of the four subpopulations were further examined at the level of cell proportions among the CD8+ cells as shown in Fig. 4. The proportion of CD28+CD57- cells (Fig. 4a) was lower among the cancer patients than the controls at all time-points, though not significantly at one month. This difference resulted from the significantly lower proportion of CD28+CD57- cells among the stage IV cancer patients compared with the controls (Fig. 4e). Corroborating the observations on the absolute cell numbers, the proportion of CD28+CD57+ cells at the 3^rd^ month was significantly higher in the stage III patients than the controls (Fig. 4f); similarly, the proportion of CD28-CD57+ cells among the stage IV cancer patients at all sampling points was significantly higher than among the control group, while the proportion at baseline was also higher than for the stage III patients (Fig. 4h). Among the control group, the baseline proportion of CD28-CD57+ cells was significantly higher than the follow-up time-points. Notably, the proportion of CD28-CD57- cells increased over the 6 months period among the cancer patients, due to the evolution of the cells among stage IV patients, which became significantly higher than the baseline at the 6^th^ month (Fig. 4c & g).

**Fig. 5:**
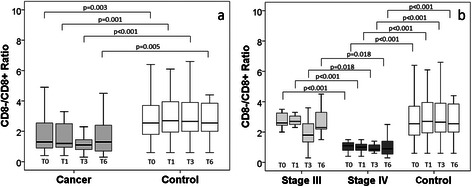
The ratio of CD8-/CD8+ T-cells among (**a**) lung cancer patients and controls, (**b**) according to cancer disease stages, at baseline (T0), 1 month (T1), 3 months (T3), and 6 months (T6)

The ratio of CD8-/CD8+ T-cells among the participants is shown in Fig. 5. The lung cancer patients had a significantly lower CD8-/CD8+ ratio than the control group at all time-points, which can be attributed to the significantly lower CD8-/CD8+ ratio among the stage IV patients at all-time points, when compared with the stage III patients and the controls respectively.

## Discussion

To provide further insight on the role of immunosenescence during cancer, variations in subpopulations of CD8+ T-cells, including the senescent CD28+CD57+ and CD28-CD57+ cells [1, 3], were followed in patients with different cancers affecting the lungs (stages III and IV) , receiving chemotherapy, over a period of 6 months. Although no clear infections were present at the time of diagnosis, the higher baseline counts of leukocytes among the cancer patients might be attributed to increased neutrophils, likely due to infectious lung processes that are often a component of advanced lung malignancies. As the decrease of the white blood cell and lymphocyte counts following chemotherapy complicates the interpretation of the results, we also took the proportional representation of the cell populations into account.

Before the onset of chemotherapy, the cancer patients presented a subpopulation profile of CD8+ T-cells associated with immunosenescence. This was evidenced by the higher level, both in absolute cell count and proportion, of the senescent and terminally differentiated, effector memory enriched CD28-CD57+ cells in the cancer patients compared to the controls. The proportion of CD28-CD57+ cells remained at the same high level during the six months follow-up in the patients. Cancer disease advancement might have played a role in the observed differences, given the higher level of CD28-CD57+ cells in the stage IV patients and the lack of difference between the stage III patients and the control group. The inverse observation was made for CD28+CD57- cells, harbouring the naïve and central memory cell populations, with lower proportions among the cancer patients, resulting mainly from the lower values among stage IV patients.

The naïve and central memory T cells have been identified as more efficient tumour-reactive T-cells than the effector/terminally differentiated effector memory cells, while the homing of T-cells to secondary lymphoid tissues is important for optimal effectiveness against tumours [35]. The CD28+CD57- cells satisfy both conditions by being enriched with naïve and central memory cells and having characteristics associated with homing to secondary lymphoid organs [32]. The lower proportion of CD28+CD57- cells observed among the stage IV patients thus appears to be particularly unfavourable. Cancer patients with advanced disease usually experience decline in naïve and central memory T-cells [48]. This might result from an ‘immune subversion force’ driving the enhanced differentiation of the naïve and central memory T-cells to less functional phenotypes, favouring the promotion of tumour growth and metastasis.

Complementing the accumulation of CD28-CD57+ cells and the decline in CD28+CD57- cells, a decreased ratio of CD8- to CD8+ cells was observed among the cancer patients compared to the controls. The decline was also most prominent among the stage IV patients. A decreased ratio of CD4+/CD8+ cells has been identified as an immunosenescence marker [4, 17]; it has been shown that CD8- T-cells are constituted mainly (>95 %) by CD4+ cells, making CD8- T-cells a workable approximation of CD4+ T cells [3, 49].

Taken together, our observations in patients with malignancies affecting the lungs bear resemblance to an IRP, which has been described as a more pronounced form of immunosenescence [6]. IRP has an unfavourable prognosis and often results in a shortened life expectancy [17, 50]. Since IRP is thought to originate from enhanced antigen exposure and persistent immune stimulation, tumour antigens might play a role in the differences in CD8+ T-cell subpopulations that we observed [21, 35]. As we have no information on the immune status of the patients prior to the cancer diagnosis, it cannot be ascertained whether the baseline differences that we observed are prior to or are a consequence of the presence of the cancers. Also, as the majority of the cancer patients were at advanced disease stages, the possible role of persistent associated infections in influencing immunosenescence might not be completely ruled out. However, our earlier report on breast cancer patients that showed strong evidence of immunosenescence, using the same indices measured here, even at the early disease stages [36], as well as reports from other groups on the association of immunosenescence with other malignancies [20–23], tend to affirm our present report on the association of cancers of the lung with immunosenescence. Our observation related to immunosenescence in peripheral blood T-cells of these cancer patients also corroborates the enhanced immunosenescence observed in peripheral blood T-cells of breast cancer patients and in T-cells isolated from the tumours [23].

Stage III patients had a an evolution of CD28+CD57+ cells that culminated in a significantly higher level and proportion at the 3^rd^ month compared with the controls. Of importance is that the 3^rd^ month corresponded to the average point of chemotherapy withdrawal among the cancer patients, even though some patients restarted chemotherapy after 6 months. A higher level of senescent CD28+CD57+ cells might be attributed to SIPS due to DNA-damaging chemotherapy and radiotherapy [24, 25]. An enhanced expression of markers of cellular senescence in T-lymphocytes has recently been shown in breast cancer patients treated with DNA-damaging agents [51]. As a corollary, we also showed the tendency of chemotherapy to induce the formation of senescent T-cells among breast cancer patients [36]. An alternative explanation could be the induction of apoptosis by chemotherapy in the more proliferating CD28+CD57- and CD28-CD57- cells, while CD28+CD57+ and CD28-CD57+ cells, due to their senescent character, would be spared [52–57]. The tendency of CD28-CD57- cells to undergo further proliferation is buttressed by its better reconstitution capacity following chemotherapy withdrawal to levels above the baseline. The faster reconstitution capacity of CD28- cells than of naïve and memory cells after DNA-damaging chemotherapy has been previously demonstrated [58]. Our present observations corroborate our earlier report on the better reconstitution capacity of CD28-CD57- cells among breast cancer patients after chemotherapy withdrawal [36]. This was not observed among CD28-CD57+ cells. Together, both reports indicate that CD28-CD57- cells might account for the higher expansion rate of CD28- cells [58], further differentiating the CD28-CD57- cells from the non-or slowly proliferating CD28-CD57+ cells, and providing *in vivo* evidence for the likely proliferation of CD28-CD57- cells.

CMV infection has been found to intensify immunosenescence in the elderly [4, 50, 59]. However, differences in immunosenescence related parameters between cancer patients and healthy controls were found not to depend on CMV seropositivity [21]. Therefore, the CMV status might not have played a significant role in the differences observed in the present study. This was buttressed by the higher age of the control subjects, and the observation of a higher degree of immunosenescence in the cancer patients than in the older control group. Immunosenescence has been shown to increase with chronological age among normal adults, even without any disease interference [3, 50, 60]. Without their pathological condition, therefore, the cancer patients would be expected to present a lower degree of immunosenescence than the normal older control group; but the reverse was observed in this study.

## Conclusions

In conclusion, the present study shows that immunosenescence and immune risk parameters appear to be more pronounced in patients with lung cancer and other malignancies affecting the lungs than in controls, and might be related to cancer disease advancement. The study also points to the possible induction of cellular senescence by DNA-damaging drugs in humans *in vivo*. The more pronounced IRP among the stage IV compared with stage III patients could provide more insight in cancer disease stages. If further explored, such differences might be useful in disease stage classification and for the selection of patients for therapy. Due to our limited sample size, we could not determine whether correlations exist between the immunosenescence status of individual patients, and their overall survival and response to therapy. Further studies will be needed to clarify these relationships.

## Abbreviations

7-AAD: 7-amino actinomycin-D
BSA-PBS: buffering solution
CD: Cisplatin & docetaxel
CDK: Cyclin dependent kinase
CE: Cisplatin & etoposide
CG: Cisplatin & gemcitabine
CMV: Cytomegalovirus
CP: Cisplatin & pemetrexed
CV: Cisplatin & vinorelbine
FITC: Fluorescein isothiocyanate
HIV: Human immunodeficiency virus
IRP: Immune risk profile
MM: Malignant mesothelioma
N: Number
NSCC: Non squamous cell carcinoma
NSCLC: Non-small cell lung cancer
PBL: Peripheral blood leukocytes
PE: R-Phycoerythrin
Q1: Lower quartile
Q3: Upper quartile
R: Radiotherapy
SCC: Squamous cell carcinoma of the lung
SCLC: Small cell lung cancer
SIPS: Stress induced premature senescence
T0: Baseline, before treatment
T1: After 1 month
T3: After 3 months
T6: After six months
